# Complete genome sequence of *Bradyrhizobium* sp. 62B, a native nitrogen-fixing rhizobium isolated from peanut nodules

**DOI:** 10.1128/mra.00928-23

**Published:** 2024-02-22

**Authors:** Fiorela Nievas, Santiago Revale, Sacha Cossovich, Emiliano Foresto, María Evangelina Carezzano, Pedro Alzari, Mariano Martínez, Mathilde Ben- Assaya, Damien Mornico, Maricel Santoro, Francisco Martínez-Abarca, Walter Giordano, Pablo Bogino

**Affiliations:** 1 Departamento de Biología Molecular, Instituto de Biotecnología Ambiental y Salud (INBIAS-CONICET), Facultad de Ciencias Exactas, Físico-Químicas y Naturales, Universidad Nacional de Río Cuarto, Río Cuarto, Córdoba, Argentina; 2 Wellcome Centre for Human Genetics, University of Oxford, Oxford, United Kingdom; 3 Unité de Microbiologie Structurale, Institut Pasteur, CNRS UMR 3528, Université de Paris, Paris, France; 4 Hub de Bioinformatique et Biostatistique - Département Biologie Computationnelle, Institut Pasteur, Paris, France; 5 Department of Biochemistry, Max Planck for Chemical Ecology, Jena, Germany; 6 Department of Plant and Soil Microbiology, Structure, Dynamics, and Function of Rhizobacterial Genomes, Estación Experimental del Zaidín-CSIC, Granada, Spain; University of Strathclyde, United Kingdom

**Keywords:** *Bradyrhizobium*

## Abstract

We present the complete genome sequence of *Bradyrhizobium* sp. 62B, a strain isolated from the root nodules of peanut plants that grow in central Argentina. The genome consists of 8.15 Mbp, distributed into a chromosome of 7.29 Mbp and a plasmid of 0.86 Mbp.

## ANNOUNCEMENT

The genus *Bradyrhizobium* (1) comprises genetically diverse rhizobia, which can establish symbiosis with different legumes (2), including peanut (*Arachis hypogaea* L.) (3–5). The isolation and characterization of native nitrogen-fixing strains may be key for the development of sustainable agriculture models (6–10).

Here, we announce the complete annotated genome of *Bradyrhizobium* sp. 62B, a native strain isolated from peanut grown in central Argentina (33°06′27″S, 64°17′60″W). The strain was isolated from a single root nodule, which was sterilized, stabbed, and aerobically cultivated on YEM agar plates (11) for 7 days at 28°C (7). After restreaking to obtain a single colony, a pure culture grown on YEM liquid for 7 days at 28°C was used to confirm the symbiotic ability on peanut plants (7), and was the source of total gDNA for sequencing. DNA was obtained either with a DNeasy Blood & Tissue kit (Qiagen, MD, USA) for Illumina sequencing (P2M platform, Institut Pasteur, France), or a Wizard HMW DNA extraction kit (Promega, WI, USA) for Oxford Nanopore Tech (Oxford Genomic Centre Services, UK). The Illumina library was prepared using a Nextera XT DNA Library Preparation kit and then sequenced on an Illumina NextSeq 500 instrument with a paired-end 150 bp read configuration. Two libraries were prepared for Nanopore sequencing: one with an Oxford Nanopore Technologies Rapid Barcoding sequencing kit (SQK-RBK004, transposase fragmentation) and the other with a Native Barcoding Genomic DNA sequencing kit (SQK-LSK109 with EXP-NBD104, mechanical fragmentation). Each library was individually sequenced in two Flongle flow cells. Data were basecalled with Guppy v4.2.2, using the high-accuracy model and the --trim_barcodes option. We obtained a combination of Illumina PE reads and Nanopore long reads (Table 1). All bioinformatic analyses were performed using default parameters unless otherwise specified. Hybrid genome assembly was performed using all the libraries together with the nf-core/bacass pipeline (commit date 2021-03-23) (12). QC filtering and trimming were performed on both read sets as part of the nf-core/bacass pipeline. The assembly resulted in two contigs that were closed by manually analyzing the overlapping ends on Geneious version 2019 2.1 (13). Replicons were rotated based on the fixstart method, with *dnaA* and *repA* as the start positions for chromosome and plasmid, respectively (14), and annotated with Prokaryotic Genome Annotation Pipeline v6.6 (15–17). The whole genome consists of one chromosome (7,289,796 bp, G + C content 64.5%) containing a single ribosomal operon and 48 tRNAs, and a repABC plasmid (859,431 bp, G + C content 60%) (18), encoding a total of 7,518 proteins across both replicons. Average nucleotide identity analysis (ANI calculator on line version) (19) revealed taxonomic identification of 62B at the genus level, being the closest genome (97.63%) *Bradyrhizobium* sp. CCBAU 53351 (NZ_CP030059.1).

**TABLE 1:** Main metric data associated with the sequenced *Bradyrhizobium* sp. 62B strain

Accession	Technology	Number of reads	Total sequencing data (Mbp)	GC content (%)	Coverage	Average length (bp)	Longest sequence (bp)	N50 (bp)
SRR17042237	Nanopore	39,581	164.5	63.61	20	4,156	48,037	7,771
SRR17042238	Nanopore	5,560	24.8	63.27	3	4,453	67,593	12,925
SRR17042239	Nanopore	10,135	53.3	63.31	7	5,257	70,559	12,787
SRR17042240	Nanopore	6,531	34.6	63.73	4	5,296	66,708	12,464
	Total Nanopore	61,807	277.2	63.54	34	4,484	70,559	8,926
SRR17042241	Illumina	6,537,438	965.1	63.7	119	151		

In contrast to other *Bradyrhizobium*, which generally harbor genes involved in symbiosis on chromosomic islands, the *Bradyrhizobium* sp. 62B genome contains them in the plasmid (20, 21). It also carries genes predicted to be related to conjugative transfer proteins and the type three secretion system (22), features related to broader host specificity and putative plasmid transfer toward other strains (23).

## Data Availability

The complete genome sequence of *Bradyrhizobium* sp. 62B is available at NCBI GenBank under accession CP092841 for the chromosome and CP092842 for the plasmid with BioProject ID PRJNA782308 and BioSample accession SAMN23371906. Raw data reads are available at NCBI’s Sequence Read Archive under accessions SRR17042237, SRR17042238, SRR17042239, SRR17042240, and SRR17042241.
